# Feasibility and acceptability of magnetic resonance imaging and electroencephalography for child neurodevelopmental research in rural Ethiopia

**DOI:** 10.3389/fpubh.2025.1551982

**Published:** 2025-07-11

**Authors:** Firehiwot Workneh, Theresa I. Chin, Kalkidan Yibeltal, Krysten North, Nebiyou Fasil, Workagegnhu Tarekegn, Betelhem Haimanot Abate, Sarem Mulugeta, Gellila Asmamaw, Atsede Teklehaimanot, Sonya V. Troller-Renfree, Sarah K. G. Jensen, Moriah E. Thomason, Terrie Inder, Charles A. Nelson, Alemayehu Worku, Anne CC Lee, Yemane Berhane

**Affiliations:** ^1^Department of Epidemiology and Biostatistics, Addis Continental Institute of Public Health, Addis Ababa, Ethiopia; ^2^Division of Biology and Medicine, Warren Alpert Medical School of Brown University, Providence, RI, United States; ^3^Department of Pediatrics, Brigham and Women’s Hospital, Boston, MA, United States; ^4^Harvard Medical School, Boston, MA, United States; ^5^Department of Reproductive Health and Population, Addis Continental Institute of Public Health, Addis Ababa, Ethiopia; ^6^Department of Global Health and Health Policy, Addis Continental Institute of Public Health, Addis Ababa, Ethiopia; ^7^Department of Nutrition and Behavioural Sciences, Addis Continental Institute of Public Health, Addis Ababa, Ethiopia; ^8^Addis Continental Institute of Public Health, Bahir Dar, Ethiopia; ^9^Addis Continental Institute of Public Health, Addis Ababa, Ethiopia; ^10^Addis Ababa University College of Health Science, Tikur Anbessa Specialized Hospital, Addis Ababa, Ethiopia; ^11^Department of Human Development, Teachers College, Columbia University, New York, NY, United States; ^12^Boston Children’s Hospital, Boston, MA, United States; ^13^Department of Child and Adolescent Psychiatry, New York University Grossman School of Medicine, New York, NY, United States; ^14^Center for Neonatal Research, Children’s Hospital of Orange County, California, CA, United States

**Keywords:** magnetic resonance imaging, electroencephalogram, acceptability, feasibility, rural, Ethiopia

## Abstract

**Background:**

Magnetic resonance imaging (MRI) and electroencephalography (EEG) are valuable tools for studying neuroanatomical and electrophysiological features of early brain development. Studies implementing neuroimaging tools in low- and middle-income countries are still rare, and there is limited data on the acceptability of such tools among rural communities. The present study explores the perceptions, feasibility, and acceptability of introducing MRI and EEG for child development research in the rural Amhara region of Ethiopia.

**Methods:**

A total of 40 in-depth interviews were conducted among community members (*n* = 24) and clinicians (*n* = 16). A semi-structured interview included four themes: (1) Baseline imaging knowledge, (2) Perceptions of MRI and EEG, (3) Facilitators and barriers to acceptability of MRI and EEG, and (4) Recommendations to improve MRI and EEG uptake. Interviews were conducted in Amharic, the local language. All interviews were transcribed verbatim to Amharic, translated into English, and double-coded. We used thematic analysis to organize data according to predefined and emerging themes.

**Results:**

Knowledge of MRI and EEG was limited, and none of the community members had previous experiences with either technology. Broadly, participants responded positively to our introductory videos showing MRI and EEG acquisition and expressed high levels of acceptability. However, participants reported concerns about possible harms related to radiation, electrical shock, and injury from MRI/EEG procedures. Those with lesser education were identified to be less accepting of MRI/EEG. In addition, several mothers expressed that consent from their husbands was necessary for their child’s participation in neurodevelopmental research. Potential logistical barriers identified included transportation challenges to the neuroimaging study sites, especially for rural-dwelling families. Creating awareness, using explanatory videos, and engaging community members and clinicians were recommended to facilitate acceptance of EEG and MRI.

**Conclusion:**

In this formative study, MRI and EEG were viewed as acceptable methods for assessing child neurodevelopment in rural areas of Ethiopia. Community members’ and clinicians’ views were impacted largely by social, religious, educational, and logistical aspects. Concerns related to MRI radiation, electrical shock, and injuries from EEG can be addressed through awareness creation and education. Engaging community leaders and healthcare providers is key to improving acceptability.

## Introduction

In low-and middle-income countries (LMICs), approximately 250 million children under 5 years of age are at risk of not achieving their full developmental potential and failing to meet expected cognitive or socio-developmental milestones (1, 2). This increased risk of developmental delay in low-income settings can affect human capital and productivity into adulthood (3). A majority of developmental delays among children in LMICs remain unidentified, precluding children from accessing necessary health services and early developmental interventions (1). Magnetic resonance imaging (MRI) and electroencephalography (EEG) can help characterize the anatomy and function of the developing brain and aid in the early identification of infants at risk for developmental delays (4, 5). These techniques are generally non-invasive, safe, and well-tolerated in children in Western contexts (5–8). However, neurodevelopmental studies implementing well-established neuroimaging techniques such as MRI and EEG have been largely restricted to high-income countries (1, 8). Historically, the use of neuroimaging methods has been limited by cost factors and the lack of access/availability in developing countries (4). Recent developments of cost-effective and portable neuroimaging devices, such as point-of-care MRI systems and portable EEG devices, are poised to benefit research in LMICs by providing a cost-effective method for assessing neurodevelopment (6–9). Thus, implementing low-cost and accessible imaging devices will aid in characterizing neurodevelopmental trajectories and identifying potential delays and may allow for early diagnosis and intervention, ultimately improving developmental outcomes (6).

The lack of familiarity with neuroimaging modalities such as MRI or EEG may lead to difficulty in implementing these neuroimaging methods in rural LMICs due to a lack of community acceptability. A study in India that examined the acceptability and feasibility of administering EEG in rural communities showed initial parental hesitancy to consent due to concern that the device could harm their children (9). Other studies in LMICs emphasized the importance of working with parents and local community members to gain acceptance when implementing novel imaging devices (10). As cultural values can influence how new technologies are understood and adopted, it is important to explore local communities’ perspectives, levels of awareness, and acceptability before introducing new technologies to ensure community buy-in and engagement. Prior to the introduction of MRI and EEG technology for a research study in a rural community in Amhara, Ethiopia (NCT06296238), we conducted a qualitative study to explore the feasibility and acceptability of neuroimaging techniques (MRI and EEG) for assessing child neurodevelopment among community members and clinicians.

## Materials and methods

We conducted semi-structured, in-depth interviews (IDI) between September and October 2022 to inform the introduction of MRI and EEG in Amhara, Ethiopia, and the development of educational materials for the Impact of Maternal Antenatal Nutrition and Infection Treatment Interventions on Longitudinal Infant Development and Growth (“LIDG”) longitudinal child follow-up study in rural Ethiopia (NCT06296238) (11). The LIDG study follows infants born to mothers enrolled in the Enhancing Nutrition and Antenatal Infection Treatment (“ENAT”) cluster randomized trial (ISRCTN15116516) (12). The LIDG study aims to examine the effects of prenatal nutrition and infection management interventions on offspring’s long-term growth and neurodevelopmental outcomes.

### Study site

This formative study was conducted in four selected health centers involved in the ENAT study in the West Gojjam and South Gondar zones (12), and two health centers and a comprehensive referral hospital in Bahir Dar, the regional capital of the Amhara region. The comprehensive referral hospital provides imaging services that include both MRI and EEG.

### Study design, participants, and sampling procedures

This study used a qualitative approach to assess MRI and EEG knowledge, perceptions, and acceptability with key informant interviews. We used a purposive sampling procedure to identify study participants. Among community members, we interviewed two mothers who had children two years or younger at each health center. We also interviewed other community members, including fathers, religious leaders, women development armies, and community administrators. Among clinicians, we interviewed health center directors, nurses, and health officers working at the pediatric outpatient department at each health center, as well as radiography technologists, pediatricians, and EEG nurses at the referral hospital. To be eligible, clinicians needed to have more than 2 years of experience working in the study area, and be currently providing child health services to communities of the selected health center, be the director of the health center, or have previous imaging experience (for hospital staff). This purposive selection of participants enabled us to gather detailed information about the feasibility and acceptability of the new technologies in rural areas. The information obtained from the IDIs helped develop a community-guided and acceptable strategy for introducing neurodevelopmental assessments, including MRI and EEG, in the rural population of Amhara.

### Participant recruitment

Written informed consent was obtained for all study participants. For those with limited literacy, a witness who was either a family member (if in attendance) or study staff read the consent form, and participants provided thumbprints to consent to their participation. We based our sample size on the principle of saturation (13). Drawing on our previous experience in this study area and population, we anticipate that a sample size of 10–15 participants per group will be sufficient to achieve both code and meaning saturation, allowing for a thorough exploration and characterization of key issues and themes (13–17).

### Data collection

A semi-structured interview guide was developed based on existing literature to capture the main themes related to community awareness, perceptions, and acceptability of MRI and EEG in research assessing child development. Clinicians were also interviewed about their prior experiences and the feasibility of implementing MRI and EEG at the health centers and the hospital. The interview guides were developed in English and translated into Amharic, the local language of the study area. Interviews were conducted face-to-face by research staff who had master’s level training in public health as well as relevant experience in qualitative data collection techniques, analysis, and write-up. Research staff were paired when conducting interviews. All IDIs took place on the premises of the selected health facilities in a private location and focused on 4 main themes. The first theme was regarding participants’ prior experience with imaging. Participants then watched a five-minute introductory video, after which they were interviewed about their perceptions of MRI and EEG (Theme 2) and the facilitators and barriers influencing their acceptability of these technologies (Theme 3). The introductory video described the benefits, potential harms, and procedures of the Hyperfine MRI machine and EEG device used for the study. The fourth theme focused on participant, recommendations to improve the uptake of MRI and EEG within their community. To minimize the burden, clinicians were interviewed at a convenient time when the patient load was lighter. With the participant’s permission, the interviews were recorded using digital audio recorders along with interview notes. The interviews took an average of 45 minutes.

### Data analysis

The interviews were transcribed verbatim in Amharic and translated into English for analysis. Data were analyzed using thematic analysis (18). Four themes were pre-defined based on specific questions developed *a priori* to examine the (1) baseline imaging knowledge, (2) perceptions of MRI and EEG (3) facilitators and barriers to acceptability of MRI and EEG, as well as (4) recommendations to improve MRI and EEG uptake. In the familiarization phase, three independent researchers (FW, KY, and TC) reviewed all full-text translations and identified codes line by line using both inductive and deductive approaches. All translations were double-coded. Microsoft Excel was used for coding, grouping themes and categories within our larger topic areas, and conducting synthesis. The results of this initial coding process were compared and agreement was reached on the overall thematic framework. Illustrative quotations were integrated with the narrative description of the study findings.

### Ethical considerations

The study was approved by the institutional review boards of Addis Continental Institute of Public Health (Addis Ababa, Ethiopia) (ACIPH/IRB/002/2022) and Massachusetts General Brigham (Boston, Massachusetts, United States) (2023P000461). Support letters were obtained from Amhara Public Health Institute (Bahir Dar, Amhara, Ethiopia), and permission was obtained from the zonal health departments, health centers, and the referral hospital. We obtained informed written consent from all the participants after providing detailed information on the purpose of the study. Participant responses, recordings, and data were kept anonymous and no names were recorded. Participants were not compensated and did not directly benefit from study participation. Participants were not directly informed of the study results, but the findings were presented at each of the study health centers.

## Results

### Participant characteristics

Our study sample (Table 1) included 24 community members (12 mothers, 6 fathers, and 6 community representatives) and 16 clinicians (two health officers, nine nurses, one EEG nurse, and two radiography technologists). Approximately 50% of the community members had completed primary education. Most clinicians had more than five years of professional experience, which may have helped them gain a deeper perspective on neuroimaging technologies.

**Table 1:** Characteristics of community members and clinicians in rural Amhara, Ethiopia.

Participant characteristics		
Community members		**n = 24** n (%)
Age	Mean age, years ± SD	30 (±8)
Education level	Primary school	11 (48)
	Secondary school	9 (39)
	Higher education	3 (13)
Marital status	Married	21 (91)
Family size	< Five	22 (96)
Number of under two children	None	3 (13)
	One	16 (70)
	Two	4 (17)
Role of participants	Mothers	12 (52)
	Fathers	6 (26)
	Other community members	5 (22)

Clinicians		n = 16 n (%)
Age	Mean age, years ± SD	32 (±4)
Marital status	Married	14 (93)
Experience	<Five years	1 (7)
	>Five years	15 (93)
Number of children under two	None	3 (13)
	One	8 (53)
	Two	4 (17)

Data reported as n (%) unless otherwise stated.

### Themes and subthemes

#### Theme 1: Baseline imaging knowledge

All community members had some prior familiarity with medical imaging, primarily X-ray and ultrasonography. CT scanning and MRI were mentioned by only four participants, all of whom had higher levels of education or resided in the regional capital with higher likelihood of being exposed to media advertisements and other sources of information (e.g., the Internet, social media). The primary sources of imaging knowledge were self-experience and from other individuals such as family members. All mothers mentioned having an ultrasound during prenatal care at nearby health facilities. Additionally, three community members mentioned personally having had an X-ray. The other community members noted that they were exposed to information related to X-rays and ultrasounds during their visits to the health centers. Whereas no community members were familiar with EEG, MRI was mentioned by two mothers and one community member, all of whom were residing in Bahir Dar.

Clinicians who work at the health centers had heard about MRI and EEG during their clinical training. However, none had observed the procedures or were aware of their availability within Ethiopia. The hospital radiography technicians had experience conducting other imaging techniques (e.g. CT, X-ray), but not MRI. Except for one nurse, all other clinicians were new to EEG procedure being conducted either at a health center or hospital.

#### Theme 2: Perceptions of MRI and EEG

After watching the introductory video, community members and clinicians were asked about their perceptions of MRI and EEG. Community members and clinicians had positive attitudes toward both of these neuroimaging methods for clinical and research use. Additionally, they expressed positive feelings and perceived neuroimaging as potentially helpful for monitoring children’s growth and development. Clinicians perceived the Hyperfine MRI as safe for infants and children given the very low magnetic field and low sound intensity, and were pleased about the young age of infant scanning allowed. Clinicians also perceived the EEG to be easier than the MRI since it would be conducted while children are awake and sitting on their mother’s lap, which was believed to minimize anxiety in the mothers. However, some concerns and misconceptions were also raised regarding the safety of MRI and EEG. In particular, community members and health center clinicians were concerned about potential exposure to radiation and risk for epilepsy with having an MRI, as well as possible electric shock for EEG. For MRI, concerns were raised about possible adverse outcomes if the baby woke up during the MRI procedure, and whether the mother would be accompanying the child during the scan. For EEG, the concerns were related to whether the cap would be worn for a long duration. Another concern shared was the treatment and management plan if any abnormalities were detected during the scans. The perceptions and concerns of community members and clinicians about MRI and EEG after the are summarized below in Table 2, along with representative quotes.

**Table 2:** Representative quotes regarding perceptions of MRI and EEG from community members and clinicians.

Perception 1 (Positive)	
To help monitor children’s development	*(MRI) “To know the growth, development, and overall health of the child – the machine is important.”* Health Center Director *(*EEG*) “To my understanding, EEG is good as it studies their brain speed and how they understand the external world.”* Pediatric Clinician
Ease of procedures	*(MRI) “It’s good and I do not think it would have a (negative) impact on the child since it is done when they are asleep and there will not be much movement, and they will not feel any pain.”* Father *(MRI) “I do not believe that a technologically advanced device has a disadvantage.”* Father *(EEG) “EEG is fairly simple: the infant does not need to be put to sleep and the procedure can be done while the child is awake and moving. The child will also be on the lap of the mother and not put in the machine. Therefore, it will lessen the mothers’ anxiety”.* Health Center Director
Safety	*(MRI) “The low magnetic field is not harmful to children.”* Pediatric Clinician *(MRI) “The Hyperfine low-field MRI is better for children because of its lower disturbance. The high-field MRI machine produces a loud sound which is not good for the ears. Thus, I think the Hyperfine is preferable.”* Radiology Technologist *(EEG) “Normally, when such kinds of imaging devices become available, they are approved by the Ethiopian Food and Drug Authority, so I do not think it has much adverse effect.”* Health Center Director
Perception 2 (Negative/Concern)	
Safety	*(MRI) “If I take a healthy child and put him under radiation, I would not know what problems it could cause in the future.”* Mother *(MRI) “There are people who suspect that these machines could cause epilepsy.”* Pediatrics Clinician *(EEG) “I have some doubts that since it is electric, it might result in some side effects. The investigation is good, but I’m afraid that it will bring some harm.”* Health Center Director *(EEG) “What is the cap on the head used for? What are these dot-like things [electrodes]? I was worried about the dots, whether they would cause injury or not.”* Community Member [Women Development Army]
Procedural concerns	*(MRI) “If the baby wakes up during the procedures, does the MRI machine slide away?”* Father *(EEG) “Does the duration of wearing the cap cause any type of problem?”* Father
Management of incidental findings	*(MRI) “If the brain development is impaired and if the baby cannot start to talk well, what will be done? Will the baby be given tablets? How will it be managed?”* Father *(EEG) “We may get children who have some disorders, so how are we going to deal with them regarding treatment?”* Health Center Director

#### Theme 3: Facilitators and barriers to acceptability of MRI and EEG

A majority of participants felt that MRI and EEG procedures would be acceptable for children and families in the community. During interviews, participants were prompted on factors that may influence the acceptability of MRI and EEG use for research on healthy children in their community. Several participants reported that individuals with lower education may be less accepting of MRI and EEG due to a limited understanding of the procedures. By contrast, several clinicians suggested that those with lower education would be more trusting of recommendations from their clinicians and thus more accepting of MRI and EEG, whereas those with higher education may be more likely to challenge the use of MRI and EEG on their healthy children for research purposes. Logistical factors, including lack of transportation and job demands, especially among rural-dwelling families, were identified as possible barriers to acceptability. Several participants also reported that mothers may require permission from their husbands prior to their children undergoing MRI or EEG. Finally, religion may also influence the acceptability of MRI and EEG among community members. Those who are religious may be less accepting of MRI and EEG due to beliefs that their child’s development is determined by God and additional investigations may not be necessary. However, religious leaders may play a role in encouraging community members, which may aid in improving the uptake of MRI and EEG. Representative quotes summarizing identified barriers and facilitators of acceptability are shown in Table 3.

**Table 3:** Representative quotes summarizing facilitators and barriers to acceptability of MRI and EEG.

Theme 3: Facilitators and barriers of MRI and EEG uptake	
Education	*“Those who lack education might reject this because they will have difficulty accepting this due to lack of mental capacity.”* Father *“Uneducated people might say ‘you will do something to my kid after putting him inside the [MRI] machine and exposing him to electricity and radiation.’ They will even say ‘I will not trust you and I will not give you my kid’.”* Community Member [Board member of the Health Center] *“The educated may challenge us more. They may say that ‘my son is healthy and does not need anything’.”* Health Center Director
Location	*“If they took the child to another location for inspection, I would not be thrilled, but mothers will be glad if their children are evaluated right where they are.”* Mother *“If it is done in larger towns; in a hospital setting then you may need a place to stay if you need to stay overnight.”* Father
Urban vs. rural dwelling	*“Rural people may not accept it because of their working time, and they may not be willing to go away and have the follow-up.”* Mother *“The farmers in the rural area are busy farming so they are not close to the information usually. And there is no responsible body to create a good understanding for them, so they may not accept it easily.”* Mother *“I would say those in the cities would have a probability of accepting this because they could have access to the media.”* Father
Role of husbands/fathers	*“My husband will accuse me of exposing the children to radiation while they are healthy.”* Mother *“As for me, I do not think a father would even send her [mother] alone, they have to go together. They have to be there.”* Mother *“It would be good if I ask his [father’s] permission.”* Mother *“There is no problem with me spending the night at Bahir Dar [location of MRI assessment], but my husband might not allow me to go there.”* Mother *“Most of the mothers who come from the rural area, if they come alone, either they want to discuss it with their older child or wait till their husbands arrive for whatever imaging type unless it is an emergency investigation; Most of the time the problem is they may request for their child or husband to come discuss and decide.”* Pediatrician
Religion	*“Religious people may not accept it, they will refuse and say God knows for us, God gives everything including the brain.”* Health Center Director *“Most of them relate it with religious issues, approaching them through religious fathers will persuade them.”* Health Center Director

#### Theme 4: Recommendation to improve MRI and EEG uptake

Participants made numerous recommendations to facilitate the community’s acceptance of MRI and EEG, revolving mainly around broadening the introductory video, community mobilization activities, and engaging clinicians and community leaders.

- *Introductory video:* Develop a comprehensive video that includes the purpose, benefits, potential risks, and side effects of MRI and EEG procedures for the child, as well as the process for result notification and follow-up care. Clear, contextually relevant examples tailored to the community’s level of understanding can improve comprehension and facilitate acceptance by the community.
- *Awareness creation activities:* Organizing health education sessions at the health center or community gatherings to educate the community using multiple approaches such as presentations, or the distribution of informational flyers, can facilitate community education. Additionally, broader dissemination of the information through local broadcasting channels may help to reach a broader population and create awareness.
- *Engagement of community leaders:* Engaging key figures in the community, including local administration and religious leaders, would enhance acceptability.
- *Engaging clinicians:* The community demonstrated a high level of trust in clinicians and it was noted that their participation in educational sessions would improve acceptance of the procedures.
- *Logistics:* Facilitation of transportation and lodging arrangements to address the needs of the family was mentioned as important to enable participation

## Discussion

This paper discusses the findings from a formative study conducted as part of the LIDG infant follow-up study to examine the first-time implementation of MRI and EEG for research in rural Ethiopia (11). Community members and clinicians had positive perceptions and high levels of acceptability, driven by a desire to learn about a child’s development. The main concerns were related to possible harms from radiation from MRI, and electrical shock and injuries from EEG. Creating community awareness will help address these concerns. Additionally, the engagement of key community members and healthcare providers would be important for improving the acceptance of these new technologies in rural areas.

Our study showed that community members were familiar with the two commonly used imaging devices, ultrasound and X-ray, but not MRI and EEG. Similar to previous studies, knowledge of ultrasound was mainly related to familiarity, as community members – especially women – mentioned prenatal care as the main source of knowledge for an ultrasound (19). Understanding previous knowledge of imaging (ultrasound and X-ray) gained through different sources helped establish a baseline for introducing new technologies in rural areas. This existing knowledge can be leveraged to build trust and address concerns about more advanced imaging through targeted educational efforts, using ultrasound and X-ray as a relatable reference to improve acceptability.

The present study highlights positive perceptions of neuroimaging research for assessing child growth and development among community members and clinicians. A similar qualitative research found that parents viewed neuroimaging positively if it centered on medical benefits and comfort (20). Despite broadly positive responses, interviews revealed possible therapeutic misconceptions, where participants mistake participation in research using MRI and EEG as clinical care. Findings emphasize previous recommendations to establish clear guidelines to address potential misconceptions by offering participants opportunities to ask questions, communicating the broader purpose of research, and emphasizing that participation in research does not substitute clinical care or confer any direct benefits (21).

The concerns and misunderstandings raised were related to the safety of the machines and fear of possible adverse effects on their children’s health. The Healthy Brain and Child Development study revealed that, even though parents were familiar with MRI, their knowledge about the safety of the procedure was minimal, with only half of the participants understanding MRI was safe for babies (22). Misperceptions that MRI would expose their children to harmful radiation were also reported in a study conducted in Nepal, where 85.5% of the study participants perceived that MRI uses harmful ionizing radiation like radiography and CT scanning (22, 23). Such misperceptions likely stemmed from the study participants’ medical imaging knowledge being limited to general imaging tools such as X-ray which are known to present risks of radiation. A study that assessed children’s and parents’ perception of MRI after examination also noted that parents felt anxious because of the limited information they received beforehand (24). Therefore, providing detailed and accurate information about the safety of MRI and EEG may help minimize these concerns (24, 25).

Several factors were identified as possible barriers to MRI and EEG. Most participants believed that those with lower education are less likely to accept MRI and EEG due to limited understanding and awareness of neuroimaging procedures. However, clinicians involved in the present study expressed that those with lower education may be more likely to trust recommendations provided by medical professionals. Together, these findings support conclusions from previous work on the importance of participant education regarding the procedures, as well as community sensitization and strengthening of local partnerships via the engagement of key community representatives to create greater awareness of MRI and EEG (21). Consistent with previous reports examining EEG acceptability in India, logistical factors such as lack of transportation may serve as a barrier to participation, particularly for families in rural areas (1). Further, communities in the rural areas are largely composed of farmers, and demands of these professions may prohibit attendance to MRI or EEG research visits. Additionally, approval from husbands was brought up as a critical factor for mothers’ ability to consent to their child’s participation in MRI and EEG. Relative to individualistic cultures in Western study settings, it is common practice for husbands to make healthcare decisions in the family-oriented cultures that are predominant in LMICs.

Engaging local stakeholders, doing formative work, and designing studies based on the formative work are crucial to understand community acceptability of MRI and EEG. Based on the findings of this formative study, we refined educational materials and the consenting protocol before study initiation. Specifically, existing introductory videos included additional comprehensive information regarding the safety of MRI and EEG on children. Additional descriptions of the misconceptions related to procedural safety were also included in the video. Leveraging these recommendations during the study, consent rates for MRI/EEG were high (>80%) for the LIDG infant follow-up study (11).

A key strength of this study is the inclusion of a diverse group of community members, including fathers, who are historically underrepresented in child development literature; community influencers, such as religious leaders; and clinicians who play essential roles in rural Ethiopian communities. This allowed us to capture detailed, personal perceptions and diverse perspectives, providing a deeper understanding of the acceptability and feasibility of MRI and EEG in rural areas. Limitations of the study include possible self-report bias. Participants may have overstated or understated their views about neuroimaging technologies due to social desirability.

## Conclusion

In conclusion, our study highlighted the overall acceptability and positive perception of MRI and EEG for neurodevelopmental assessment in children in rural Ethiopian communities. The perception and acceptability of MRI and EEG among community members and clinicians were influenced by various social, religious, educational, and logistical factors. While most participants expressed positive views of MRI and EEG, concerns about possible harms and side effects–including MRI radiation, electric shock from EEG, and discomfort from EEG caps–were notable. Addressing these concerns with targeted awareness-creation and educational activities is important. It is recommended that future neuroimaging research in LMIC prioritize engaging community leaders and healthcare providers to build trust and enhance the acceptability of these technologies in similar settings.

## Data Availability

The raw data supporting the conclusions of this article will be made available by the authors, without undue reservation.
